# Sir Arthur Stanley's Visit. Who Has Been "Done"?

**Published:** 1918-06-29

**Authors:** 


### NURSING AFFAIRS IN IRELAND.

### SIR ARTHUR STANLEY'S VISIT. WHO HAS BEEN "DONE"?

THE Irish situation is unique, and the English reader cannot be expected to understand. He never understands. He is dull-witted and slow, whilst Irish thought and Irish wit are subtle. Ireland is proud; Ireland is a land of artists and philosophers, and the English are mere shopkeepers. The concentrated policy of England, from the King down, has ever been to plunder poor Pat, who built up England's mighty Empire—to "do him in the eye," and having extracted from him all that he possesses, and a little more, to rejoice. Cruel, gormandising, tyrannising England!

In Ireland the thing that tickles our fancy most is that the Englishman cannot understand. Which is perfectly true. He cannot. Neither can we, which of course is a secret that we keep to ourselves. Some of us want England to set us up as a republic with an army, a navy, and a Diplomatic Service. We want to make treaties with France and Spain and Patagonia, whither we may from time to time export some potatoes and butter, and perhaps a few Limerick hams on special conditions. And alas! as the vernacular has it, "the polis won't let us." *Hinc illæ lacrimæ.*

Personally, I am a litle curious as to what Sir Arthur Stanley thinks of us in his heart of hearts. Sir Arthur came amongst us more or less like an apparition, and disappeared. It was his first visit to Ireland since the war broke out, and he stayed three days, during which we all wore our Sunday clothes and looked good. But we reckon we impressed him, and gave him a few things to think about! We fulfilled our destiny by convincing him that we are a peculiar people, and therefore we have little cause for complaint. We have shown him that aureola of mystery which is our distinguishing feature, whose significance no man here or elsewhere can comprehend.

Sir Arthur Stanley's idea, of course, was crude. His object was to appeal to the British nation on behalf of British nurses, or, as up to a particular date we here described it, as an attempt to "insult" the nurse by offering her "charity," and thereby sap and undermine her independence. So far as we were concerned, we felt that Erin's honour and Erin's pride in the nursing department, at all events, were at stake, and later we scented a plot whereby, through some cunning artifice, the Irish nurse was to be plundered. And so accordingly we held our proud heads high and unfurled the banner of Sinn Fein—"Ourselves alone." Sir Arthur Stanley pleaded the wider hope, but in vain. And then, to the astonishment of everybody, he quietly agreed. From this shock we have not yet quite recovered, and now we don't quite know where we are. But we are beginning to feel that we are probably outside the gate. In the meantime we have abandoned the "charity" stunt, and we are free to "insult" the nurse as much as we please, whatever Mrs. Bedford Fenwick and Company, Limited, may say to the contrary. We are going full steam ahead to collect what we can. We have appropriated Sir Arthur Stanley's idea, made it our own, and politely informed him that his connection with it there ends. It had never before occurred to our brilliant intellects that the Irish nurse lacked for aught, although we knew that out of her poverty she paid twenty, thirty, and even fifty guineas for her training, and sometimes ended her days in the workhouse.

The question now arises—"Who has been done"? We remain in the glorious position of being independent. We may collect and gather together and distribute to our heart's content. It has come to us as a revelation that we had the power to do this wonderful thing, and our national vanity is satisfied at being able to establish ourselves in a unique position. The other little bit of the Empire does not matter. We might, perhaps, have been more gratified if Sir Arthur Stanley had taken umbrage. The annoying thing was that he only smiled, patted us on the back, and told us what wonderful people we are! I want to know, however, who has been "done," and in this connection may I recall a nursing incident of long ago? The dull-witted English tried and succeeded in passing a Midwives Act, and the promoters of the Bill begged for Irish co-operation. The answer was "Certainly not. We have the Rotunda Hospital: we know all about midwifery, and it is you who are in a back street."

And it took us full fifteen years of blinking before we opened our eyes to the truth, despite the fact that we are the people, and when we speak no dog dare bark.

There is only one person about whom I have a pang of regret—the voiceless toiler who never saw Sir Arthur Stanley, and who, mayhap, never heard of him. The ladies with the "Ourselves alone" banner never consult the nurse. And this nurse can make the figure of zero just as well as I can, representing the spiritual or financial benefits that she has received week by week and year by year from this centre of faction. What of this forgotten work-weary toiler? She is Irish. Is she to have no opportunity of saying whether she prefers the heaven that the Irish Nurses' Association have made and are making for her, or, instead, to enrol with her sisters in the Imperial Order of British Nurses, and share their fortune and their fate? She may be ignorant, but she is sufficiently knowing to be able to calculate how much in hard and vulgar cash, directly or indirectly, she owes to the leaders who, according to their own account, have been championing her cause for the past quarter of a century. I am genuinely sorry for this nurse, because, after all, she is the one and only person really concerned. And I am inclined to the belief that it is she who has been "done."

